# Improved first-pass effect in acute stroke thrombectomy using Solitaire-X compared to Solitaire-FR

**DOI:** 10.3389/fneur.2023.1215349

**Published:** 2023-10-19

**Authors:** Ron Biederko, Asaf Honig, Ksenia Shabad, Yair Zlotnik, Gal Ben-Arie, Farouq Alguayn, Ilan Shelef, Anat Horev

**Affiliations:** ^1^Clinical Research Center, Soroka University Medical Center, Beer Sheva, Israel; ^2^Neurology Department, Soroka University Medical Center, Beer Sheva, Israel; ^3^Faculty of Health Sciences, Ben Gurion University of the Negev, Beer Sheva, Israel; ^4^Radiology Institute, Soroka University Medical Center, Beer Sheva, Israel; ^5^Department of Neurosurgery, Soroka University Medical Center, Beer Sheva, Israel

**Keywords:** acute ischemic stroke, endovascular thrombectomy, first pass effect, pusher wire, large vessel occlusion, Solitaire-X, Solitaire-FR, Solumbra technique

## Abstract

**Background:**

In acute ischemic stroke (AIS), successful endovascular thrombectomy (EVT) of large vessel occlusion (LVO) necessitates the most suited device. Solitaire-X has longer and larger diameter pusher wires than Solitaire-FR.

As the role of a larger pusher-wire diameter is uncertain, we aim to compare procedural, clinical, and radiological outcomes for AIS patients undergoing EVT using either type of Solitaire device. Procedures were performed using the Solumbra technique, which combines a large-bore aspiration catheter with a stentriever. The primary outcome was to compare rates of successful first-pass recanalization (defined as TICI 2b/3 score). The secondary objectives were procedural (rates of successful recanalization), clinical (post-procedural NIHSS and days of hospitalization), and radiological (post-procedural ASPECT score and hemorrhagic transformation) outcome measures.

**Design:**

Consecutive AIS patients undergoing EVT for LVO were recruited into a prospective multicenter database at our academic center. We have used Solitaire-FR until October 2020 and Solitaire-X ever since. We retrospectively analyzed our prospective consecutive registry. Included in our analysis are patients undergoing EVT using Solitaire only; patients with tandem lesions or underlying stenosis requiring emergent stenting during the procedure were excluded. The cohort of patients treated with Solitaire-X was compared with a cohort consisting of the most recent consecutive cases undergoing EVT with the Solitaire-FR.

**Results:**

A total of 182 (71.9 ± 14, 61% male patients) AIS patients were included in the analysis with both groups (*n* = 91 each) sharing similar demographic characteristics, premorbid conditions, and stroke characteristics (time from symptom-onset, NIHSS, ASPECTS, occlusion site, and rates of intravenous-tPA treatment). The Solitaire-X group had a higher rate of first-pass recanalization (65.9% vs. 50.5%, *p* = 0.049). On 24-h post-procedural head-CT, the Solitaire-X group had higher ASPECT scores (6.51 ± 2.9 vs. 5.49 ± 3.4, *p* = 0.042) and lower post-procedural average bleeding volumes (0.67 ± 2.1 vs. 1.20 ± 3.4 mL, *p* = 0.041). The Solitaire-X group had shorter duration of hospitalization (16.6 ± 13.1 days vs. 25.1 ± 23.2, *p* = 0.033). On multivariate analysis, using Solitaire-X was the sole independent predictor of first-pass recanalization (OR 2.17, 95% CI 1.12–4.26, *p* = 0.023).

**Conclusion:**

In our study, the use of the Stentriever-X with a larger pusher-wire diameter was associated with a higher likelihood of first-pass effect and improved procedural, clinical, and radiological outcomes in AIS patients.

## 1. Introduction

Over the years, stroke thrombectomy devices and techniques have undergone significant evolution. The main devices are stentrievers and aspiration catheters. They are used in a range of techniques, including aspiration alone (ADAPT), stentriever alone, or a combination of both aspiration and stentriever (Solumbra). Additionally, every procedure may be performed with or without a balloon guide catheter (BGC). Multiple variations of technical approaches have been developed to further enhance the efficacy and safety of the procedure (1–3). Several studies have discussed the differences between available stentrievers and the qualities required to improve the device’s effectiveness (4, 5). Other studies have suggested that the length and size of the stentriever itself may predict the first-pass effect (6, 7).

The efficacy of aspiration is increased when a large-bore catheter is positioned proximal to the clot and as straight as possible to increase aspiration force. Several techniques have been devised to improve aspiration efficacy, including the use of a BGC and straightening the catheter with a balloon (8). The Solumbra technique combines the use of a large-bore aspiration catheter with a stentriever (Solitaire, Medtronic, Minneapolis, MN, United States) and consequently provides several potential synergistic effects. One of them is the straightening of the aspiration catheter with the stent retriever wire, thereby potentially improving the catheter trackability, thus allowing improved positioning and aspiration force. This technique may have a special advantage in cases with challenging cervico-cerebral anatomy (1).

In our medical center, the Solumbra technique is the preferred option for most MCA occlusion cases. The efficacy of the Solitaire-FR has been shown extensively in previous studies (9–11). When Solitaire-X became available, we switched to using it exclusively instead of Solitaire-FR since we hypothesized that it would allow faster procedures. Both devices are stentrievers that share an identical stent structure, but there are three key differences between them. First, the Solitaire-X has a smaller profile that allows for delivery through a 21-inch microcatheter, whereas the Solitaire FR requires a 27-inch microcatheter for delivery of the 6 mm device. Second, the pusher wire of the Solitaire-X is longer, measuring 200 mm, as opposed to the 180 mm pusher wire of the Solitaire-FR. Finally, the Solitaire-X has a larger diameter of 0.0018″, whereas the Solitaire FR has a diameter of 0.0016″.

In this study, we aimed to compare the procedural and clinical outcomes of Solitaire-FR to Solitaire-X. As the main difference between the devices is the pusher wire length and diameter, we believe that this comparison will enable us to possibly extrapolate the effectiveness of stentrievers with longer and larger diameter pusher wires. To the best of our knowledge, our study is the first to investigate the effect of pusher wire diameter on the first-pass effect.

## 2. Methods

### 2.1. Study population

Consecutive AIS patients undergoing EVT for LVO were recruited into a prospective multicenter database of our academic tertiary-level center. All EVT procedures at our medical center are performed by the same neurointerventionalist, without a trainee, using the Solitaire-FR until October 2020 and Solitaire-X afterward. We aimed to compare procedural, clinical, and radiological outcomes between the groups treated using each device. Our prespecified primary objective was to compare the rate of first-pass successful recanalization (defined as modified thrombolysis in cerebral infarction (mTICI) 2b − 3 score). Our prespecified secondary objectives were procedural (rates of successful recanalization), clinical (post-procedural NIHSS, and discharge modified Rankin score (mRS), number of hospitalization days and 90-day mortality), and radiological (post-procedural ASPECT score and hemorrhagic transformation volume) outcome measures. In this study, AIS patients >18 years old who underwent EVT within 24 h of symptom onset were included. Patients with occlusions in the carotid terminus, M1 or proximal M2 of the middle cerebral artery (MCA), or the basilar artery were eligible for the study if they had a National Institutes of Health Stroke Scale (NIHSS) score of ≥6 or had a major neurological deficit (e.g., aphasia). Patients presenting in the early time window (<6 h from onset or last seen normal) were chosen for EVT based on an Alberta Stroke Program Early CT Score (ASPECTS) of ≥6. For these patients, perfusion imaging was not mandatory. However, in the late time window (>6 h and < 24 h from last seen normal), we applied radiological selection criteria using perfusion CT studies. The CT perfusion criteria used in our institution were similar to the radiological eligibility criteria used in the Defuse-3 trial (12): initial infarct volume (ischemic core) <70 mL, a ratio of volume of ischemic tissue (penumbra) to infarction >1.8, and an absolute volume of potentially reversible ischemia >15 mL. The use of intravenous thrombolysis (IV-tPA) was not a contraindication for participation in the study.

AIS patients were excluded from the study when Solitaire was not the first device used, they had tandem lesions, there was underlying stenosis requiring emergent stenting during the procedure, or they underwent thrombectomy using techniques other than Solumbra.

The cohort of patients treated with Solitaire-X was compared with a cohort consisting of the most recent consecutive cases undergoing EVT with the Solitaire-FR.

### 2.2. Endovascular treatment

All procedures were performed under general anesthesia with femoral artery access. The procedure involved placing a guide catheter (NeuronMax, Penumbra, Alameda, CA, United States) in the cervical arterial segment of interest after visualizing an intraluminal thrombus angiographically. The guide catheter was advanced as far distally as possible in the vertebral artery (VA) or internal carotid artery (ICA) while ensuring safety. Next, a delivery microcatheter (Headway 21 or 27, Microvention, Aliso Viejo, CA, United States; Phenom 21, Medtronic) was passed through a large-bore aspiration catheter (Sofia 5 or 6, Microvention) in a triaxial fashion over a microwire (Synchro 14 Stryker, Kalamazoo, MI, United States). The aspiration catheter of the largest possible size that could be accommodated by the vessel was selected, and the microcatheter was advanced over the guidewire through the thrombus while positioning the aspiration catheter just proximal to the thrombus. A Solitaire device was deployed through the microcatheter from distal to proximal across the occlusion site. Catheter size was selected according to labeling indications. A 40-mm length stent was used in all cases. In the following large-vessel occlusions (ICA, M1 or proximal M2 MCA segments, and basilar artery of dominant VA), a 6 mm width Solitaire stent was used. In other, small-diameter vessels, a 4 mm width Solitaire stent was used. The delivery microcatheter was removed before thrombus extraction to allow for increased cross-sectional luminal area for aspiration, and suction was applied by connecting the aspiration catheter to a penumbra aspiration pump. The stent retriever was then withdrawn into the large-bore aspiration catheter either during local aspiration or as a complete assembly with the aspiration catheter. Subsequent angiographic runs were performed to evaluate revascularization, and the procedure was repeated as necessary. Further revascularization attempts were performed if TICI2b − 3 was not achieved. If no recanalization was achieved after three attempts, a different stent retriever was used.

After successful recanalization was achieved, groin closure with Angioseal was performed and the patient was admitted to the stroke unit or general ICU unit, depending on bed availability. Prior to leaving the angiography suite, all patients underwent a non-contrast head CT scan (NCCT). Repeated NCCT was performed 24-h post-procedure prior to initiating antiplatelet or anticoagulation treatment.

### 2.3. Data collection

To ensure accuracy, a neurologist reviewed time metrics and clinical and demographic data and also verified clinical outcome measures. Patient history and demographic data were collected from the electronic medical file. In order to ensure a similar economic level, we added a personal economic score according to the Israeli Central Bureau of Statistics (CBS).

Data on procedural variables, including mTICI score at the end of the procedure and the number of passes needed to achieve the best possible recanalization, were reviewed by the neurointerventionalist. Radiological data were collected by a specialized radiologist blinded to the clinical scenario. Both volumetric assessment of the hemorrhagic transformation (HT) and classification of the ASPECTS were performed using the 24-h post-procedure NCCT.

### 2.4. Ethical considerations

The study adhered to IRB guidelines (SOR-0133-21) with a waiver of informed consent.

### 2.5. Statistical analysis

Quantitative data were summarized using means and standard deviations for normally distributed variables, medians and ranges for other quantitative variables, and percentages for qualitative variables. The Mann–Whitney test was used to compare the time from the first symptoms to the procedure, the number of passes and mTICI, and mRS and NIHSS scores at discharge for the two groups. The rate of procedural complications, such as bleeding during the procedure and bleeding on CT on the next day, and death at discharge were compared between the study groups using the Pearson chi-square test. Multivariable analysis was conducted using logistic regression to model predictors for first-pass recanalization. Odds ratios (OR) and 95% confidence intervals (CIs) were estimated for potential risk factors, adjusting for potential confounders based on their clinical and statistical significance. Statistical significance was set at a two-sided value of p of <0.05 corresponding to a 95% confidence interval. Reported value of ps were rounded to three decimals. Statistical analyses were performed using IBM SPSS software version 25 or higher (Chicago, IL, United States) and R-studio version 4.1.1 (R-foundation).

## 3. Results

Between 2018 and 2022, 590 AIS patients underwent EVT procedures. Among them, 408 AIS patients were excluded from the study, including 15 in whom the EVT procedure began with a device not included in the study and 393 with either tandem lesions or underlying stenosis requiring emergent stenting. A total of 182 patients (91 from each group) met the study inclusion criteria. The Solitaire-X group shared similar demographic and baseline characteristics with the Solitaire-FR group apart from a higher prevalence of renal disease (18.7% vs. 7.7%, *p* = 0.049, Table 1). Stroke characteristics, including time from symptom onset to procedure, admission NIHSS and ASPECTS, and LVO site were comparable for the groups, and similar percentages of patients received bridging IV-tPA prior to the procedure (22% vs. 20.9%, *p* = 1.000).

**Table 1:** Demographic and pre-procedural clinical features.

		Solitaire FR (N = 91)	Solitaire X (N = 91)	*p* value
Age, years				
Mean ± SD		71.4 ± 13.5	72.4 ± 14.7	0.487
Median (min; max)		74.0 (25.6;90.9)	74.8(6.95;97.6)	
Sex, male – *n* (%)		58 (63.7)	53 (58.2)	0.543
Number of children				
Mean ± SD		4.5 ± 3.8	3.5 ± 2.9	0.092
Median (min; max)		3.0(0;21)	3.0(0;12)	
CBS social state score				
Mean ± SD		4.0 ± 2.5	3.77 ± 2.1	0.573
Median (min; max)		4.0 (0;10)	4.0 (0;9)	
Family status *n* (%)	Married	53 (58.2)	50 (54.9)	0.529
	Widowed	27 (29.7)	23 (25.3)	
	Divorced	7 (7.7)	10 (11.0)	
	Single	2 (2.2)	5 (5.5)	
Smoking *n* (%)		18 (19.8)	18 (19.8)	0.610
Myocardial infarction *n* (%)		13 (14.3)	17 (18.7)	0.549
Previous stroke *n* (%)		31 (34.1)	30 (33.0)	1.000
Diabetes *n* (%)		35 (38.5)	29 (31.9)	0.438
Chronic renal failure *n* (%)		7 (7.7)	17 (18.7)	**0.049**
Any malignancy *n* (%)		8 (8.8)	9 (9.9)	1.000
From symptom onset to ER admission, min				
Mean ± SD		288 ± 232	225 ± 226	0.657
Median (min; max)		147(13;1,120)	136(26;847)	
From symptoms to groin puncture, min				
Mean ± SD		387 ± 248	363 ± 243	0.426
Median (min; max)		303(124;1,390)	275(105;1,140)	
ASPECT score pre-procedure				
Mean ± SD		8.5 ± 1.5	8.3 ± 1.5	0.330
Median (min; max)		8.0 (4;10)	8.0 (1;10)	
NIHSS score pre-procedure				
Mean ± SD		15.6 ± 5.1	14.4 ± 4.6	0.132
Median (min; max)		16.0 (3;28)	15.0 (4;23)	
MRS score pre-procedure				
Mean ± SD		0.7 ± 1.1	0.8 ± 1.1	0.560
Median (min; max)		0.0 (0;4)	0.0 (0;4)	
Vessel occlusion site *n* (%)	M1	45 (49.5)	44 (48.4)	0.696
	M2	21 (23.1)	18 (19.8)	
	ICA terminus	19 (20.9)	24 (26.4)	
	BA	6 (6.6)	5 (5.5)	
Intravenous thrombolysis *n* (%)		19 (20.9)	20 (22.0)	1.000

Values represent number of patients unless otherwise stated. Statistically significant *p* values are marked in bold.

The Solitaire-X group had a higher rate of first-pass recanalization (65.9% vs. 50.5%, *p* = 0.049). In order to achieve successful recanalization, AIS patients who failed to recanalize after the first pass underwent additional Solitaire passes. In each Solitaire group, there were seven patients who failed three attempts to recanalize with Solitaire, and attempts were then made using other devices. Ultimately, rates of successful recanalization were similar between the two cohorts (98.9% vs. 96.7%, *p* = 0.613), and the higher first-pass effect of the Solitaire-X cohort did not translate into a different post-procedural NIHSS score (7.03 ± 5.2 vs. 7.8 ± 5.8, p = 0.6) or a different pre-to-post-procedure improvement in the NIHSS score (7.4 ± 4.9 vs. 7.8 ± 5.4, *p* = 0.7). Nevertheless, on 24-h post-procedural NCCT, the Solitaire-X group had a higher ASPECTS (6.51 ± 2.9 vs. 5.49 ± 3.4, *p* = 0.042). The Solitaire-FR group had a double rate of post-procedural hemorrhagic transformation (29.7% vs. 15.4%, *p* = 0.021) with a higher average bleeding volume (1.20 ± 3.4 vs. 0.67 ± 2.1 mL, *p* = 0.041), and the Solitaire-X group had shorter duration of hospitalization (16.6 ± 13.1 vs. 25.1 ± 23.2, *p* = 0.033) (see Table 2).

**Table 2:** Procedure and post-procedural characteristics and patients’ outcomes by the study group.

	Solitaire-FR (N = 91)	Solitaire-X (N = 91)	*p* value
From first symptoms to recanalization, min			
Mean ± SD	431 ± 252	405 ± 243	0.411
Median (min; max)	345(151;1,430)	320(122;1,160)	
Successful first-pass recanalization *n* (%)	46 (50.5)	60 (65.9)	**0.049**
Number of passes (dichotomized)			
1–3 passes	83 (91.2)	83 (91.2)	1.000
4 and above passes	7 (7.7)	7 (7.7)	
Number of passes			
1	46 (50.5)	60 (65.9)	0.230
2	27 (29.7)	16 (17.6)	
3	10 (11.0)	7 (7.7)	
4	2 (2.2)	1 (1.1)	
Multiple passes	5 (5.5)	6 (6.6)	
Number of passes, continuous			
Mean ± SD	1.8 ± 1.09	1.6 ± 1.1	0.068
Median (min; max)	1.0 (1;5)	1.0 (1;5)	
Successful recanalization *n* (%)	90 (98.9)	88 (96.7)	0.613
Total procedure time, min			
Mean ± SD	45.1 ± 36.2	40.8 ± 33.4	0.260
Median (min; max)	34.0 (13;184)	31.0 (12;188)	
NIHSS score post-procedure			
Mean ± SD	7.80 ± 5.8	7.03 ± 5.2	0.430
Median (min; max)	7.5 (0;23)	5.0 (0;19)	
ASPECT score post-procedure			
Mean ± SD	5.49 ± 3.4	6.51 ± 2.9	**0.042**
Median (min; max)	6.0 (0;10)	7.0 (0;10)	
Hemorrhagic transformation post-procedure *n* (%)	27 (29.7)	14 (15.4)	**0.021**
Hemorrhage volume mL			
Mean ± SD	1.20 ± 3.4	0.67 ± 2.1	**0.041**
Median (min; max)	0.0 (0;24.0)	0.0 (0;11.7)	
Hospitalizations length, days			
Mean ± SD	25.1 ± 23.2	16.6 ± 13.1	**0.033**
Median (min; max)	16.0 (2;102)	13.0 (3;84)	
Number of repeated hospitalizations			
Mean ± SD	0.85 ± 1.8	0.11 ± 0.5	**<0.001**
Median (min; max)	0.0 (0;9)	0.0 (0;4)	
mRS score upon discharge			
Mean ± SD	3.89 ± 1.8	3.93 ± 1.6	0.931
Median (min; max)	4.0 (0;6)	4.0 (0;6)	
90-day-mortality *n* (%)	18 (19.8)	14 (15.4)	0.559

Values represent number of patients unless otherwise stated. Statistically significant *p* values are marked in bold.

In a univariate analysis, the use of Solitaire-X (66% vs. 50.5%, *p* = 0.049) and time from symptom onset to groin puncture were the only predictors of first-pass recanalization. Notably, the site of vessel occlusion did not affect the rate of first-pass recanalization. Moreover, on multivariate analysis (Table 3), using Solitaire-X was the sole independent predictor of first-pass recanalization (OR 2.17, 95% CI 1.12–4.26, *p* = 0.023).

**Table 3:** Multivariate analysis for predictors of first-pass recanalization.

	OR	95% CI	*p* value
Solitaire X	2.17	1.12–4.26	0.02
Time from symptom onset to groin puncture, min	1.00	0.99–1.02	0.20

OR, Odds ratio, CI, Confidence interval NA, Not available.

## 4. Discussion

Both Solitaire devices had excellent efficacy and safety profiles for EVT in the current study. The Solumbra thrombectomy technique, which combines the use of stent retriever entrapment of thrombus and aspiration, has been shown to improve the chances of achieving a first-pass effect over aspiration or stent retriever as a standalone technique (13, 14).

Notably, patients in the cohort managed with Solitaire-X had higher rates of successful first-pass recanalization. Solitaire-X is differentiated from Solitaire-FR mainly by the larger diameter of its pusher wire. We propose that this feature enhances the aspiration effect and thereby improves the ability of the Solitaire-X to achieve the first-pass effect.

Delivering large-bore aspiration catheters intracranially to the thrombus can be challenging due to vessel tortuosity, underlying intracranial atherosclerosis, or even the anatomical position, for example, at the point where the ophthalmic artery originates from the carotid siphon. Bernava et al. showed that the efficacy of thrombectomy is related to the angle of interaction between the aspiration catheter and the clot and emphasized the importance of positioning the aspiration catheter tip against the proximal part of the clot (15). Kang et al. also supported the importance of aspiration catheter tip contact with the clot and discussed the need to improve the ability to advance large-bore catheters through tortuous vessels (14). The larger pusher wire may help to straighten the microcatheter more effectively, thus improving the trackability of the aspiration catheter and enabling it to reach the optimal position for aspiration. Furthermore, enhanced straightening and overcoming kinks in the aspiration catheter likely increases the aspiration force, leading to better procedural outcomes (16). Consequently, the Solitaire-X cohort had higher rates of successful first-pass recanalization. We assume that this achievement led to radiological evidence of a reduced volume of ischemic tissue, as evidenced by better post-procedural ASPECTS in patients from the Solitaire-X cohort.

The Solitaire-X group had a lower rate of hemorrhagic transformation and smaller hemorrhagic volumes 24-h after thrombectomy. The smaller profile of the Solitaire-X may allow swifter navigation to the occlusion site with less trauma to the vessel wall. Furthermore, smaller ischemic volumes contribute to smaller volumes of hemorrhagic transformation.

There are other available stentrievers with a 0.18″ pusher wire, such as the Neva (Vasalio, Nashville, TN, United States) and Trevo ProVue (Stryker). It is highly likely that our results can be extrapolated to all devices with a 0.18″ pusher wire, but this should be confirmed in other studies. Another difference between Solitaire-FR and Solitaire-X is the length of the wire. We assume that the length of the device can possibly affect the procedural outcomes indirectly by affecting the operator’s comfort in using it.

In conclusion, this study found that the use of the Solitaire-X, which has a larger stentriever pusher wire diameter and smaller profile compared to Solitaire-FR, was associated with a greater likelihood of first-pass effect and improved radiological outcomes in acute stroke patients undergoing mechanical thrombectomy using the Solumbra technique.

This study has several limitations. First, it is a retrospective study, and several variables were not routinely included in our institutional prospective database. For instance, it lacks a full 90-day follow-up. Second, the number of included patients is relatively small, and further large-scale, randomized controlled studies are needed to reinforce our findings. Additionally, as all interventions were performed by a single endovascular neurointerventionalist a possible improvement curve may be attributed to the more recent procedures. However, the physician has performed over 3,000 stroke thrombectomies in the past decade, thus limiting such effects. Finally, the mTICI score was adjudicated by the sole neurointerventionalist possibly introducing bias.

## Data availability statement

The datasets presented in this article are not readily available because of ethical and privacy restrictions. Requests to access the datasets should be directed to the corresponding authors.

## Ethics statement

The studies involving humans were approved by the Soroka Medical Center Review Board. The studies were conducted in accordance with the local legislation and institutional requirements. Written informed consent for participation was not required from the participants or the participants' legal guardians/next of kin in accordance with the national legislation and institutional requirements.

## Author contributions

AsH: writing—original and final draft, validation, formal analysis, and visualization. AnH: conceptualization, methodology, writing—review and editing, and supervision. RB: statistical analysis. AnH, KS, FA, RB, YZ, GB-A, IS, and AsH: data curation. All authors contributed to the article and approved the submitted version.
